# Advancing public health leadership through culturally centered and responsive research mentorship training in Nigeria

**DOI:** 10.3389/fpubh.2025.1611853

**Published:** 2025-12-16

**Authors:** Rifkatu Reng, Manmak Mamven, Fatima Kyari, Elizabeth N. Christian, Anthony Orji, Roseline Abah, Nanna Ripiye, Gregory Erhabor, Mark D. Huffman, Dike Ojji, Leah C. Neubauer, Lisa R. Hirschorn

**Affiliations:** 1 University of Abuja, Abuja, Nigeria; 2 Feinberg School of Medicine, Northwestern University, Chicago, IL, United States; 3 Washington University in St. Louis, St. Louis, MO, United States; 4 The George Institute for Global Health, Sydney, NSW, Australia; 5 School of Public Health, University of Michigan, Ann Arbor, MI, United States

**Keywords:** cardiovascular disease, Nigeria, mentorship, training, Fogarty

## Abstract

Effective leadership in research and public health is critical to strengthen evidence generation and translation into policy and practice. Mentorship in research and public health is critical to develop leaders, but mentorship training is often *ad hoc* and may not be effective, particularly when it does not reflect local cultural and academic contextual realities. The Cardiovascular Research Training in Nigeria (CeRTIN) program, a funded partnership between the University of Abuja, Northwestern University, and Washington University in St. Louis is designed to increase cardiovascular research capacity and leadership of Nigerian investigators, including through mentorship training. This paper describes the development, implementation, and evaluation of a culturally-centered research mentorship training program designed to strengthen mentorship knowledge, capacity, and skills among current and future research leaders. This hybrid workshop, co-developed by faculty in Nigeria and the US using adult learning principles, was conducted in April 2024 with early/midcareer academics, professionals, and mentorship leaders (*N* = 65). Forty four participants completed post-workshops surveys. Results demonstrate high degrees of agreement with the workshop’s utility, relevancy to the cultural context, and a valuable investment of time. Participants were most engaged during group discussions and interactive sessions which mirrored real-world examples. Significant improvements were reported in self-reported confidence and knowledge in key areas of mentorship. The value and sustainability of culturally responsive approaches to research and public health leadership development through mentoring is critical to plan, execute, and sustain research to improve health in Nigeria and beyond.

## Introduction

### Need for a new generation of global health leaders in research

Effective leadership in research and public health is critical to strengthen and use evidence for decision making to bridge the gap between knowledge and delivery of interventions known to save lives and reduce suffering. However, strategies are needed to develop current and future leaders who will address disparities and emerging needs whether due to pandemics, aging populations, climate change, and the growing burden of disease. The current and emerging challenges in the global landscape of public health crises call for a new generation of leaders who can lead the design, implementation, dissemination, and translation of evidence into routine policy and practice in their settings.

Among the leading global health challenges, the burden of non-communicable, chronic diseases including cardiovascular disease pose a threat to the advances made in extending lifespan and reducing morbidity over the last few decades. In Nigeria there is a critical need for locally designed and led research in clinical trials, implementation science and policy development to address disease burden and improve health care delivery (1, 2). This research should empower leaders who can utilize evidence to enact decisions on how to design effective policy and implementation for better prevention, diagnosis, and management of non-communicable, chronic diseases (3).

Culturally responsive and culturally aware mentoring are key elements of mentoring. Developing a culturally responsive approach for public health leadership development through mentoring is a valuable and sustainable strategy to address challenges. This approach empowers mentors with the skills, knowledge and networks necessary to create lasting positive changes. Culturally-aware mentoring (CAM) is an evidence-based initiative to enhance mentors’ ability to effectively address cultural diversity matters in their research mentoring relationships. It involves practices in which mentors recognize their own culturally shaped beliefs, perceptions, and judgments and are cognizant of cultural differences and similarities between themselves and their mentees (4–6). CAM inspires mentors to understand, appreciate, and incorporate the cultural backgrounds, values, and beliefs of their mentees into the mentoring relationship.

Advancing Mentorship to Promote Supportive Research Environments.

The capacity of public health leaders and researchers in many low- and middle-income countries (LMIC) is still emerging, although capacity building work is underway through training and mentorship (7). This need to mentor researchers in LMIC to reduce inequities has been discussed as a “pivotal part of succession planning in global health” (8). Mentorship is critical to supporting researchers throughout their career, and particularly as the new generation of leaders in research emerges (9, 10). However, the understanding of how mentorship can be made more effective in LMICs is still relatively new. Effective mentoring relationships provide the building blocks for competent leaders to navigate complex dynamics with the flexibility and conscientiousness needed to improve the health of global populations (11). A 2019 report on the Fogarty-funded mentoring workshops focused on training mid and senior-level researchers on tools and techniques of effective mentoring was found to be effective in enhancing skills and capacity in researchers and promoting supportive research environments in LMIC (5, 12).

The Cardiovascular Research Training in Nigeria (CeRTIN) program, a collaboration between the University of Abuja in Nigeria, Northwestern University, and Washington University in St. Louis, was funded by National Institutes of Health Fogarty International Center to increase the research capacity of Nigerian investigators in cardiovascular implementation research, clinical trials, and patient-centered outcomes research. The objectives of the overall program include developing new leaders in research, strengthening mentorship of and by current and future leaders, and building coursework with public health faculty at the University of Abuja. The CeRTIN program investigators recognized the need to develop mentorship capacity in the current and future researchers to ensure they acquire the skills and competencies necessary to mentor junior researchers. In the CeRTIN program, each trainee is assigned a mentor from the University of Abuja or another leading Nigeria academic institution and one or two mentors from the US institutions (triad model). This approach is supported by a commitment to developing and building capacity for current and future mentors in cardiovascular research in the University of Abuja and other institutions in Nigeria.

## Pedagogical framework(s), pedagogical principles, and competencies/standards underlying the educational activity

While mentoring is a key component of development of research leaders, training in mentorship is not always available, systematic, nor effective, particularly when it does not reflect local cultural and academic context realities. These collaborative efforts built on the existing mentorship model and an initial training on mentorship and facilitation (Goldberg, et al., 2023). The workshop was co-developed by faculty in Nigeria and the US and was conducted in a hybrid format incorporating adult learning principles with mentorship leaders at the University of Abuja and wider region.

### Short-course format to build capacity

Short-course training as workshops is a core strategy from the Fogarty International Center and was a key component of the CeRTIN program, including PhD and master’s candidates, post-doctoral fellows, and junior faculty at the University of Abuja. The goals of the short-course approach involved teaching new methodologies and concepts to adult learners to build knowledge, skills, and interest in progressing as researchers and mentors. The short-term mentorship workshop was designed to provide more senior investigators at the University of Abuja and strategic partners with skills and competencies necessary to mentor junior researchers and to develop a research workforce capable of conducting high-impact cardiovascular health research at independent and team levels. The team enacted this core strategy during the first 2 years of the project to teach in the core methodological areas including IS, clinical trials and PCOR as well as mentorship and facilitation. Additional emphasis will be placed on key elements in the mentoring ecosystem including nurturing a culture of institutional mentoring, competency-based frameworks, peer mentoring, cohort learning, and learner-centered pedagogy.

### Educational framework for mentorship workshop

The CeRTIN program’s educational framework was guided by adult learning principles, Bloom’s Taxonomy, and the Kirkpatrick Evaluation Model (13–15). The training focused on building the skills and achieving results to enrich mentee and mentor capacity. This focus was on practical skills, collaborative learning, and continuous development that exist at the core of this training program which aims to create confident, competent, and committed mentors who can effectively guide and support their mentees. Practical application and skill development over theoretical lectures were emphasized. Bloom’s Taxonomy was modified for adult training to enable participants to build knowledge and skills, while clarifying what they need to know and why it is important.

## Learning environment (setting, students, faculty); learning objectives; pedagogical format

### Workshop development and design

The University of Abuja-led workshop was built on an initial two-day workshop held on 25–26 of April 2024. This workshop agenda was co-developed from evidence-based practices in mentorship and an initial mentorship workshop hosted by team members years prior (16). The team expanded mentorship training within the program through the co-creation of the workshop content and format. The process placed emphasis on leveraging cultural wisdom and expertise and adapting existing mentorship tools and principles to reflect the local context and culture. Local examples and leaders emphasized the cultural landscape for academics in Nigeria. The workshop was largely designed to be in person but opportunities to attend the lectures and participate in large group discussions were offered for attendees unable to come in person. A full detailed agenda highlighting workshop topics, duration and sequencing is found in Supplementary Table 4.

### Training approach

The training approach leveraged principles of adult learning by equipping mentors with knowledge and skills that they could readily use to be effective. Instead of focusing on the theoretical aspects, the emphasis was placed on participatory learning approaches, practical application in professional work settings, and knowledge and skill acquisition and development. Key sessions topics are detailed in the agenda (Supplementary Appendix). Example topics included the following: mentoring frameworks & definitions, effective mentoring in local contexts, mentee panel, setting expectations, and elder reflections on mentoring in local contexts. Several key pedagogical strategies were used, including interactive workshops, group discussions, role-playing, and case studies. Used together, the environment focused on active learning, peer learning, and collaboration and fostered community among the mentors and mentees. The active learning focus promoted key mentoring skill-building activities including active listening, effective communication, goal setting, and providing constructive feedback. Mentors and mentees were provided with a toolkit of resources, including templates, guides, and articles to support their practice. The workshop was limited to 2 days because of resource constraints.

### Trainers and facilitators

Three physician-scientists from the University of Abuja who also serve as mentors and key personnel in the CeRTIN program led the training. Workshop presenters included leaders in mentorship from Nigeria and South Africa as well as current mentors (Nigeria and US) and mentees from the CeRTIN program. In addition, current trainees from CeRTIN participated in the workshop and reflected on their own experiences as mentees and emerging mentors in a panel session.

### Evaluation framework

The Kirkpatrick Model is an internationally recognized tool for evaluating and analyzing the results of educational, training and learning programs (15). It consists of four levels of evaluation: *Reaction, Learning, Behavior, and Results*. Areas of the evaluation addressed multiple levels of the Kirkpatrick Framework including Level 1 (Reaction), Level 2 (Learning), and Level 3 (Behavior). Level 1 measures participants’ immediate perceptions and satisfaction with the training. Level 2 measures how well participants acquired the desired knowledge, skills, and confidence in the core domains (communication, constructive feedback, expectations, goal setting, strategy development, independence, & connection). Level 3 assessed participants’ intentions and evidence of knowledge application into practice. Participants completed pre- and post-workshop surveys using Likert scales, knowledge assessments, and open-ended questions.

### Data analysis approach

Data was entered into REDCap managed by the University of Abuja. Descriptive statistics were used for demographics and quantitative responses. A rapid, pragmatic approach guided the thematic analysis of qualitative data, enabling an examination of the dynamic context and systems within the training environment, and informing recommendations for meaningful and actionable improvements (17, 18). To ensure anonymity a participant generated code was used to link the pre and post session surveys. Missing data were excluded.

## Results

Sixty-five individuals attended the workshop on one or both days, including 12 virtual and 53 in person. The workshop attendees included a broad spectrum of early and mid-career medical professionals in the cardiovascular and related professions from Nigerian universities, hospitals, and primary healthcare centers alongside professional medical associations like the Nigeria Hypertension Society, Nigeria Medical Association, pharmacists’ associations, and individuals from ministries and other policymakers. Of the 65 persons who attended, 45 completed the pre-survey, 44 completed the post-survey, and 37 participants were matched with pre and post surveys.

### Evaluation respondents

#### Pre-survey results

Of attendees, 45 (69%) completed the pre-test which included demographics. Table 1 shows the baseline characteristics of the participants who responded to the pre. Among 45 respondents (57% female), 13 (36%) self-identified as both mentors/mentees and 24/37(64%) self-identified as mentees. Nearly half (16, 43%) reported that they had led two or more research projects. Regarding the highest parent education, two thirds (25, 67%) of parents had tertiary education. Of those with pre-workshop, 22 (49%) were female, representing four states in Nigeria. one third (32.9%) were relatively new to cardiovascular health, 57.7% had never led a research project and 58% had never written a grant. Two thirds were in the role of a mentee only (64%), 6.7% were mentor only and 29% were in both roles.

**Table 1:** Mentorship workshop pre-survey respondent characteristics (*N* = 45).

Characteristic	N (%) or median (IQR)
Gender	
Female	22 (49%)
Male	23 (51%)
Current state	
Abia	1 (2.2%)
Abuja/FTC	42 (93%)
Enugu	1 (2.2%)
Osun	1 (2.2%)
Highest education level of parent	
Primary or less	11 (24%)
Secondary	4 (8.9%)
Tertiary	30 (67%)
Experience in cardiovascular health	
New	11 (24%)
<1 year	4 (8.9%)
1–5 years	17 (38%)
> 5 years	13 (29%)
Research experience	
None	2 (4.4%)
I have worked on projects but never led a project	24 (53%)
I have led 1 or 2 projects	11 (24%)
I have led more than 2 projects	8 (18%)
Manuscript publishing experience	
No	16 (36%)
Yes, as a coauthor only	13 (29%)
yes as a first author and coauthor	15 (33%)
yes as a senior author and first or coauthor	1 (2.2%)
Number of publications if published (median)	6.00 (2.00, 19.25)
Research grant experience	
No	26 (58%)
Yes as part of a team	15 (33%)
Yes as the lead/principle investigator	4 (8.9%)
Current role	
Mentee	29 (64%)
Mentor	3 (6.7%)
Both	13 (29%)

#### Post-survey results

Forty-four (67.6%) participants completed the post workshop evaluation which included questions on Kirkpatrick Level 1 (reaction) and Level 2 (learning) (Table 2). All participants agreed or strongly agreed on the value, organization and delivery of the workshop. Importantly, 98% agreed or strongly agreed that the workshop facilitators were sensitive to differences between the mentoring context in Nigeria and the US, and 86% reported that the workshop was relevant to the work and cultural context in Nigeria. Most (86%) reported that the workshop was relevant to the work and cultural context and was a valuable investment of time (Level 1, Reaction). Significant improvements were self-reported confidence and knowledge in key areas of mentorship (Level 2, Learning) and that the tools will be useful for them as mentors and mentees in the future (Level 3, Behavior).

**Table 2:** Mentorship workshop post survey responses (*N* = 44).

Prompt	Mean (SD)	Agree or strongly agree
5 point rating from 1 = strongly disagree to 5 = strongly agree except where noted		
Kirkpatrick 1 (reaction)		
I enjoyed the workshop	4.9 (0.3)	100%
The workshop was a valuable investment of my time.	5.0 (0.1)	100%
The workshop facilitators were sensitive to differences between the mentoring context in Nigeria and the US.	4.8 (0.5)	98%
There was sufficient time for discussion during the workshop.	4.6 (0.5)	98%
There were enough opportunities for participants to participate actively in the workshop	4.7 (0.5)	98%
The workshop facilitators provided valuable feedback during the workshop	4.7 (0.4)	100%
The workshop was relevant to the work and cultural context in Nigeria.	4.4 (0.8)	86%
The workshop was relevant to the particular research context that I work in, i.e., clinical, laboratory, public health etc.	4.5 (0.7)	95%
Kirkpatrick level 2 (knowledge, skills)		
I have a clearer understanding of what my role as a mentor is.	4.7 (0.5)	100%
My overall confidence as a mentor has increased.^2^	5.8 (1.0)	–
Kirkpatrick level 3 (use)		
The tools shared during the workshop will be useful for mentors.	4.8 (0.4)	98%
The tools shared in the workshop will be useful for mentees.	4.7 (0.6)	95%

^1^1 = Strongly Disagree, 2 = Disagree, 3 = Neither agree nor disagree, 4 = Agree, 5 = Strongly Agree. ^2^Based on 7-point rating.

#### Matched pre and post survey results

**Figure 1:**
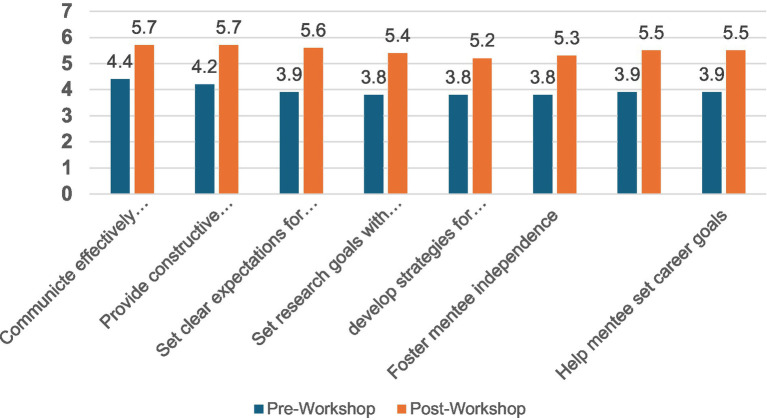
Change in mean scores for knowledge and skills following mentorship workshop among individuals who responded to pre and post workshop surveys. All *p* < 0.001.

Thirty-seven participants (82.2% of those completing the pre-survey) completed both pre and post-workshop evaluation, which focused on Kirkpatrick Level 2 (knowledge and skills). There was a significant increase in self-rated skills in all areas measured (*p* < 0.001) (Figure 1). The highest self-rated skills included communication (communicating effectiveness, providing constructive feedback), followed by setting expectations, career goals, and how to account for difference in background with their mentees. No differences were seen in change based on sex, years of mentorship or research experience, or parental education (see Figure 1).

#### Qualitative post workshop responses

The open-ended questions assessed three levels of Bloom’s taxonomy (Table 3). Level 1 (Reaction) examined aspects of learner engagement, enjoyment, and workshop design. Level 2 (Learning) examined what types of learning occurred. Level 3 (Behavior) examined how participants plan to apply what they learned from the training. Key themes in each area are detailed below. Supplementary Table 4 includes key themes and representative quotes.

**Table 3:** Post mentorship workshop open ended responses.

Level 1 (reaction): How did participants respond to the training?	
Theme	Quotes
Many opportunities for engagement and participation	*“All throughout the sessions, we were given a lot of opportunities to engage and ask questions*.” *“When we were meant to assess our skills and talk about our rating. I found it engaging and the template given was very helpful in helping me identify specific areas I need to work on.”*
Practical examples and real-world applications	*“During the group activities because of the contributions. And, during Prof Erabor’s presentation because of the many life experiences he shares.”* *“Professor Gregory because it involves a lot of practical examples in Nigeria.”*
Extended duration to enhance learning	*Spending 3 days would make participants internalize the information better*
Enhanced diversity and inclusivity for broader engagement	*“Increase the number of participants and [extending] invitation[s] to senior consultants who may need retraining on mentorship.”* *“Expanding to involve faculty from different parts of Nigeria and cascade to institutions and organizations for the benefit of more.”*
Enhance training structure	*“Have these trainings regularly, and integrated in structured learning programme,”* *“The workshop can be inculcated into curriculums.* *“Send materials ahead of the workshop so people can reflect more on it before the day.”* *“As a way of practice and to enhance continuous learning, participants could be assigned to mentors/mentees to help us build on what has been taught.”*

Level 2 (learning): How much did participants learn from the training and have their skills improved?	
Theme	Quotes
Enhanced understanding and confidence	*“This workshop has really increased my confidence level and understanding about Mentor-Mentee relationship”* *“It has helped me see some gaps in my mentoring process. I have understood how active the process is.”*
Effective communication	*“I have learnt how to be patient with my mentors and mentees. I have learnt how to give feedback, not necessarily opinion. I have learnt to factor cultural backgrounds in my relationship with my mentees. I have learnt to ensure regular follow up and not give a gap.”*
Providing constructive feedback	*“The importance of timely feedback to mentees and the top ten tips to giving feedback,”* *“Give better feedback to students and mentees and not opinions.”*
Effective goal-setting and use of Individual Development Plan (IDP)	*“This workshop will help me address the challenges I faced as a [mentee]. I’ve learned to write out my IDP and set clear goals and expectations for myself.”* *“It will help me to clearly define my goal as a mentor and also learn how to follow up with the goals”*
The role of mentorship in success	*“As a mentor, be patient with your mentees, guide them with relevant information and resources, show them available opportunities for career growth, etc.”* *“I will also encourage the professional bodies to have mentorship added to our school curricula from foundation to the post-doctoral level”*
Commitment to advance personal and professional development	*“I plan to do a lot of self-reflection, write out my IDP and have meetings with my mentor.”* *“I will self-audit, prune my life of avoidable distractions, set smart goals, set steps to achieve the goals, and periodically review the goals.”*

Level 3 (behavior): How will participants apply what they learned from the training?	
Theme	Quotes
Intent to apply learnings to practice	*“I will use all the tools and knowledge gained to enhance my mentorship skills and be a better mentor.”* *“I will use this as a road map to a successful mentoring relationship.”*
Enhanced career development through individual development plans	*“Fill this IDP form to give structure to my path to career progression.”* *“Plan to do regular evaluation of my IDP.”*
Increased proactivity in research and professional development	*“Build a strong network with people of other professions.”* *“I intend to start a departmental research group.”*
Strengthening mentor-mentee relationships	*“I will begin effective communication with my Mentor for career development.”* *“Make mentorship in my department a MUST and encourage other senior colleagues to properly mentor junior colleagues.”*

#### Level 1 (reaction): How did participants respond to the training?

Participants were most engaged during group discussions and interactive sessions. Many participants felt engaged during the entire workshop, and other participants felt engaged during the role plays. The lectures and presentations were identified as the most engaging when they included realistic and included practical examples and life experiences. Participants enjoyed the group discussions and interactive sessions since everyone contributed, and many felt engaged throughout the workshop due to the numerous opportunities to ask questions. The role plays were cited as being interactive and entertaining. Most of the respondents reported that they felt engaged in the mentoring workshop. However, some participants expressed a preference for physical interactions over virtual presentations, and one participant was unfamiliar with Individual Development Plans (IDP) which was addressed in follow-up communications to ensure it was clear. An example IDP is found in Supplementary Table 5. Overall, key themes included:

*Many opportunities for engagement and participation*. Throughout the workshop, participants felt most engaged in the opportunities presented in the lectures, presentations, group discussions, and interactive sessions.

*Practical examples and real-world applications*. Another positive theme that emerged out of the workshop was the use of practical activities for a hands-on learning experience. Many participants expressed that workshop activities and presentations felt realistic to them. The practical relevance of presentations delivered by professors seemed to be well-received by participants and enhanced the learning experience.

*Extended duration to enhance learning*. Multiple participants suggested making the workshop three days instead of two days. Having more time for group discussion and learning was reported to be more beneficial for future learning.

*Disengagement during the virtual sessions*. Three participants reported that they felt the least engaged in the mentoring workshop during the virtual sessions, as they prefer interactive [in-person] sessions. This can inform future workshop programming to prioritize in-person sessions over virtual ones.

*Enhanced diversity and inclusivity for broader engagement*. Several participants expressed a desire for a broader and more diverse group of faculty and attendees, emphasizing ways to expand the workshop’s reach, inclusivity, and interdisciplinary engagement across dimensions of age, experience, institutions and regional parts of Nigeria.

*Enhance training structure*. Participants shared feedback to enhance the training structure in three areas: more regular, structured trainings, materials to be shared in advance, and incorporating mentor-mentee matching. Some participants expressed their desire for formal mentor-mentee connections to be facilitated within the workshop setting.

#### Level 2 (learning): How much did participants learn from the training and have their skills improved?

Overall, participants left the workshop with a greater understanding of the mentor-mentee relationship, including maintaining effective mentor-mentee relationship dynamics for success, stating that they would share communication strategies and mentoring techniques. Mentors learned how to provide constructive feedback to mentees for continuous improvement. The workshop taught practical applications through the identification of goal setting and individual development plans (IDPs) as key parts of career and personal development. Responses also cited improved communication styles, increased confidence, and advancing personal and professional development in achieving objectives. The need for collaboration and peer support was also emphasized.

Key themes included:

*Enhanced understanding and confidence*. Participants reported overall increased understanding of effective mentoring dynamics and greater confidence in applying workshop concepts.

*Effective communication*. Participants highlighted the importance of refining communication skills to achieve better outcomes within their mentoring relationships. Several participants cited a newfound understanding of providing constructive feedback rather than opinion to their mentors and mentees.

*Providing constructive feedback*: Participants learned both the importance of constructive feedback and how to convey feedback to mentees, as self-reported.

*Effective goal-setting, including the use of Individual Development Plan (IDP):* Participants remarked that the workshop emphasized the relevance of structure, goal-setting, and using individual development plans. Participants reported greater confidence in applying these skills to overcome challenges, such as time management and following up on goals.

*The role of mentorship in success.* Participants reported the importance of the mentor-mentee relationship was a key takeaway from the workshop. Many are committed to sharing effective strategies on creating a relationship.

*Commitment to advance personal and professional development*. Participants expressed a renewed commitment to self-improvement and growth in their personal and professional lives through learning, reflection, collaboration and peer support. The responses reflect a workshop influence on participants to grow and improve in every aspect of their lives by setting goals and creating more intentionality.

#### Level 3 (behavior): How will participant apply what they learned from the training?

Overall, participants reported that creating and utilizing IDPs are important in furthering their goals and career development. Participants remarked on being more proactive in research, including building professional networks and collaborating with peers in different fields through networking. There was an emphasis on strengthening mentor-mentee relationships by finding mentors and creating more structure, communication, and interaction within the relationship. Additional steps included self-improvement through consistent reflection and intentionality. Key themes included:

*Intent to apply learnings to practice*. Participants expressed commitment to apply the workshop learnings in practice.

*Personal development*. Another emerging theme was the workshop's influence on participants’ personal development, self-improvement, and career advancement. Participants indicated that workshop learnings provided them greater clarity and direction in their professional and personal journey.

*Enhanced career development through Individual Development Plans (IDP)*: Many participants stated they would create and utilize Individual Development Plans (IDP) in their respective careers because of the workshop. This highlights the importance participants placed on using IDPs for structured professional development.

*Increased proactivity in research and professional development*. The workshop helped participants realize their desire to participate more in research and seek out collaboration with peers. Responses indicate motivation to advance their research careers and professional networking.

*Strengthening mentor-mentee relationships*. Participants spoke about the benefits of a strong mentor-mentee relationship and action steps to achieve a beneficial one for themselves, mentees, mentors and departments.

## Discussion on the practical implications, objectives, and lessons learned

This workshop aimed to advance public health leadership in Nigeria through culturally centered and responsive mentorship training in research. Led by local mentorship leaders, the program focused on empowering mentors to guide mentees while fostering their independence and leadership skills. The findings, based on quantitative and qualitative data, indicate the program’s success in achieving its objectives. The workshop attendees represented a diverse demographic including gender, age, workplace, culture and ethnicity, including a broad spectrum of early and mid-career professionals in cardiovascular and related professions. Most had little experience with leadership in research activities which highlights the need for capacity building for beginning and mid-level attendees. While research mentorship training has had broader implementation in several institutions in high income countries – including evidence of changing both practice and perspectives - it has not been as well developed and implemented in Nigeria (5, 19).

This workshop successfully achieved many outcomes including enhancing effective communication, constructive feedback mechanism, goal-settling in their research and career, setting expectations about mentee/mentor relationships, and mentee independence, supporting the strengthening of leadership in public health and research including in the next generation.

The evaluation, which focused on Kirkpatrick Level 2 (knowledge and skills), demonstrated an increase in self-rated skills in all areas measured. The highest self-rated skills were communicating effectiveness, providing constructive feedback and setting expectations and career goals. Attendees demonstrated self-reported increased capacity in very important areas with reported increased confidence in effective communication with mentors, learning how to clearly express themselves while actively listening, and adapting their approach to mentee needs making transfer of knowledge and skills possible. The skill of increased ability to meet on regular basis will promote mentee growth and development in all aspects of research. Participants reported a deeper understanding of ethical principles and best practices in mentoring, such as being empathic and compassionate while paying attention to differences across gender and religious backgrounds. Participant responses demonstrate their ability to ensure a positive and supportive environment in a culturally sensitive manner.

There was generally an increased motivation and commitment to mentoring, providing a greater sense of purpose and satisfaction in their mentoring roles. The training provided mentors with the tools and resources they need to be successful. Mentors learnt how to work collaboratively with mentees in setting the research goals and in identifying potential challenges in knowledge and skill gaps while developing strategies for their mentees’ success. Guiding mentees toward self-reliance and maintaining independence was another crucial skill that was learned, thus empowering them to take ownership of their research and career goals as this will promote their growth and development. Having successful mentees function as group leaders when appropriate can assist with aspects like work/life balance and time management. Skills on helping mentees identify potential funding and networking opportunities were learned.

### Practical implications

Our training approach designed to strengthen public health and research leadership to address the growing burden of NCDs in Nigeria highlights the value of culturally responsive approaches to global health leadership development that also include attention to adult learning principles, pedagogy, and evaluation. The key findings from the evaluation provide further details on the success of integrating core practices including cultural responsiveness, culturally aware mentoring, and sharing elder wisdom across continents, cultures, and generations of faculty are core to our approach.

Most participants agreed that the workshop facilitators were sensitive to differences between the mentoring context in Nigeria and the US; 86% remarked that the workshop was relevant to the work and cultural context in Nigeria. This workshop stressed the need to address cultural sensitivity while addressing the unique needs of both parties within the Nigerian context. This we envisage will facilitate stronger relationships, with trust in supportive, inclusive and safe environments. Similar work by Byars-Winston found high value and satisfaction with culturally aware mentor training, reporting gains in personal cultural awareness and cultural skills, and increased intentions and confidence to address cultural diversity in their mentoring (4).

Culturally sensitive mentors and wisdom from elders are key elements of our collective approach. It is essential in Nigeria, where diverse ethnic groups and cultural practices exist, that a culturally sensitive mentor would be aware of this and adapt their communication style accordingly. While the workshop emphasized practical, skill-based learning, several lectures were included as they were led by senior-level, elder members of the community. Cultural leaders as lecturers shared vast mentoring experiences in a culturally sensitive manner, particularly attuned to gender and age. Some cultural values shared were mutual respect, not only for mentors, but persons who are older and more experienced. For example, in Nigeria it is culturally appropriate for older individuals to be addressed using titles and not with first names. A focus on gendered examples from the Nigerian academic context highlighted the need to understand values and beliefs around cultural communication styles. This involved using culturally relevant examples to explain issues and concepts. Specific time was devoted to navigating cultural barriers such as language, enhancing inclusiveness, and providing culturally sensitive feedback (20).

### Lessons learned

Overall, participants reported that they found the training valuable and impactful; the training equipped them to provide better mentorship and address the mentees’ needs. Mentees reported feelings of confidence and empowerment and appreciated the support and focused guidance on research and leadership skills. The training team learned that culturally responsive mentoring is critical to the success of young scientists of diverse backgrounds (20). Thus, the importance of ongoing support for both the mentors and mentees cannot be overemphasized. Both reiterate the need for continuous training and opportunities to interact and network to strengthen their capabilities. Mentorship programs- whether new or recurring - benefit deeply from formal and well-structured evaluation frameworks and methods (12, 21).

While formal mentorship in low and middle-income country (LMIC) institutions has grown, literature on the evaluation of these programs remains limited (21). Further, few consensus evaluation metrics exist in high-income countries that assess mentorship at institution levels (21). Chi et al.’s recommendation for ongoing, systemic evaluation to monitor progress, programmatic gaps, quality improvement, resource justification, and forecast future directions align with the results of this Nigeria-based work. Specific focus on the stage and maturity of these programs is also important (21). Further attention to various types of formative, summative, process and outcome evaluation frameworks hold potential for understanding the role that various mentorship components play across the globe.

These findings suggest that this approach to mentorship training has the potential to significantly contribute to strengthening the public health research capacity in Nigeria. To ensure widespread adoption and to maximize the impact and sustainability of the programs, the University of Abuja based institution has commenced a mentorship program within the institution in collaboration with the Institute of Advanced Medical Research and Training and the Faculty of Clinical Sciences. It will be crucial to fund and scale up to other institutions and regions in Nigeria. The cross-institutional and cultural team agrees that there is a need to integrate this type of responsive mentorship training into existing public health and other research training programs. Success demands a network and platform for both mentors and mentees to connect, support, and share their program experiences (19, 22, 23).

A policy framework can contribute to long-term program success by acknowledging the importance of investing in future scientists and in research enabling environments. A sustainable mentorship network can also be encouraged by linking those currently mentored with an upward view to gradually step into full mentoring thereby reducing the burden of the limited number of mentors in LMIC (23, 24).

### Limitations of mentorship programs

This mentorship program, while exciting and promising, is not without challenges similar to those identified by Rosenberg, et al. (25). Securing adequate funding for future programming is vital for the commencement and sustainability of the program. Finding qualified mentors who are crucial for the program can be difficult because mentors need to have protected time, relevant experience and skills, and a commitment to effectively guide mentees (26, 27). Several assumptions that mentorship must be hierarchical or that mentors may be senior-level may hinder valuable peer mentorship opportunities (10, 28). Further examination of mentorship programs across disciplines, ranks and settings can aid in expanding understandings of the various success and challenge elements.

## Data Availability

The raw data supporting the conclusions of this article will be made available by the authors, without undue reservation.

## References

[1] Adeloye D Owolabi EO Ojji DB Auta A Dewan MT Olanrewaju TO . Prevalence, awareness, treatment, and control of hypertension in Nigeria in 1995 and 2020: a systematic analysis of current evidence. J Clin Hypertens. (2021) 23:963–77. doi: 10.1111/jch.14220, 33600078 PMC8678849

[2] Nelson IO. Management of hypertension in Nigeria: the barriers and challenges. J Cardiol Cardiovasc Med. (2021) 6:023–5. doi: 10.29328/journal.jccm.1001112

[3] Angell B Sanuade O Adetifa IM Okeke IN Adamu AL Aliyu MH . Population health outcomes in Nigeria compared with other west African countries, 1998–2019: a systematic analysis for the global burden of disease study. Lancet. (2022) 399:1117–29. doi: 10.1016/S0140-6736(21)02722-7, 35303469 PMC8943279

[4] Byars-Winston A Womack VY Butz AR McGee R Quinn SC Utzerath E . Pilot study of an intervention to increase cultural awareness in research mentoring: implications for diversifying the scientific workforce. J Clin Transl Sci. (2018) 2:86–94. doi: 10.1017/cts.2018.25, 30338131 PMC6191051

[5] Pfund C House SC Asquith P Fleming MF Buhr KA Burnham EL . Training mentors of clinical and translational research scholars: a randomized controlled trial. Acad Med. (2014) 89:774–82. doi: 10.1097/ACM.0000000000000218, 24667509 PMC4121731

[6] Han I Onchwari A. Development and implementation of a culturally responsive mentoring program for faculty and staff of color. Interdiscip J Partnersh Stud. (2018) 5:3. doi: 10.24926/ijps.v5i2.1006

[7] Hamer DH Hansoti B Prabhakaran D Huffman MD Nxumalo N Fox MP . Global health research mentoring competencies for individuals and institutions in low- and middle-income countries. Am J Trop Med Hyg. (2019) 100:15–9. doi: 10.4269/ajtmh.18-0558, 30430976 PMC6329357

[8] Ngcamu BS. Succession planning and leadership development in a faculty of health sciences. Glob J Health Sci. (2019) 11:101–1. doi: 10.5539/gjhs.v11n11p101

[9] Hamelin AM Paradis G. Population health intervention research training: the value of public health internships and mentorship. Public Health Rev. (2018) 39:1–13. doi: 10.1186/s40985-018-0084-9, 29619272 PMC5879914

[10] Ng’oda M Gatheru PM Oyeyemi O Busienei P Karugu CH Mugo S . Mentorship in health research institutions in Africa: a systematic review of approaches, benefits, successes, gaps and challenges. PLoS Glob Public Health. (2024) 4:e0003314. doi: 10.1371/journal.pgph.0003314 39312559 PMC11419371

[11] Rodríguez DC Jessani NS Zunt J Ardila-Gómez S Muwanguzi PA Atanga SN . Experiential learning and mentorship in global health leadership programs: capturing lessons from across the globe. Ann Glob Health. (2021) 87:61. doi: 10.5334/aogh.3194, 34307064 PMC8284496

[12] Gandhi M Raj T Fernandez R Rispel L Nxumalo N Lescano AG . Mentoring the mentors: implementation and evaluation of four Fogarty-sponsored mentoring training workshops in low-and middle-income countries. Am J Trop Med Hyg. (2018) 100:20. doi: 10.4269/ajtmh.18-0559 PMC6329359 30430977

[13] Brundage D. H. MacKeracher D. (1980). Adult learning principles and their application to program planning. Toronto: Ontario Department of Education.

[14] Grebin N Grabovska S Karkovska R Vovk A. Applying Benjamin bloom's taxonomy ideas in adult learning. J Educ Cult Soc. (2020) 11:61–72. doi: 10.15503/jecs2020.1.61.72

[15] Kirkpatrick JD Kirkpatrick WK. Kirkpatrick's four levels of training evaluation Association for Talent Development (2016).

[16] Goldberg BB Mbugi EV Kyari F Woods SE Balandya E Drane D . Training in the art and science of facilitation to scale research mentor training in low and middle income countries. Front Educ (Lausanne). (2023) 8:1270480. doi: 10.3389/feduc.2023.1270480, 38846335 PMC11155035

[17] Hall JN. Pragmatism, evidence, and mixed methods evaluation. N Dir Eval. (2013) 2013:15–26. doi: 10.1002/ev.20054

[18] Patton MQ. Enhancing the quality and credibility of qualitative analysis. Health Serv Res. (1999) 34:1189. 10591279 PMC1089059

[19] Cole DC Johnson N Mejia R McCullough H Turcotte-Tremblay AM Barnoya J . Mentoring health researchers globally: diverse experiences, programmes, challenges and responses. Glob Public Health. (2016) 11:1093–108. doi: 10.1080/17441692.2015.1057091, 26234691 PMC5020346

[20] Montgomery BL Mondisa JL Packard BWL. Promoting the cultivation and sustainability of mentoring ecosystems: results from a multi-institutional study. Mentoring & Tutoring: Partnership in Learning. (2024) 32:596–617. doi: 10.1080/13611267.2024.2389051

[21] Chi BH Belizan JM Blas MM Chuang A Wilson M Chibwesha CJ . Evaluating academic mentorship programs in low- and middle-income country institutions: proposed framework and metrics. Am J Trop Med Hyg. (2019) 100:36–41. doi: 10.4269/ajtmh.18-0561, 30430978 PMC6329356

[22] DeCastro R Sambuco D Ubel PA Stewart A Jagsi R. Mentor networks in academic medicine: moving beyond a dyadic conception of mentoring for junior faculty researchers. Acad Med. (2013) 88:488–96. doi: 10.1097/ACM.0b013e318285d302, 23425990 PMC3610810

[23] Lescano AG Cohen CR Raj T Rispel L Garcia PJ Zunt JR . Strengthening mentoring in low-and middle-income countries to advance global health research: an overview. Am J Trop Med Hyg. (2018) 100:3. doi: 10.4269/ajtmh.18-0556 PMC6329352 30430982

[24] Hamid M Rasheed MA. A new path to mentorship for emerging global health leaders in low-income and middle-income countries. Lancet Glob Health. (2022) 10:e946–8. doi: 10.1016/S2214-109X(22)00230-3, 35714639

[25] Rosenberg JL Holincheck N Fernández K Dreyfus BW Wardere F Stehle S . Role of mentorship, career conceptualization, and leadership in developing women’s physics identity and belonging. Phys Rev Phys Educ Res. (2024) 20:010114. doi: 10.1103/PhysRevPhysEducRes.20.010114

[26] Straus SE Johnson MO Marquez C Feldman MD. Characteristics of successful and failed mentoring relationships: a qualitative study across two academic health centers. Acad Med. (2013) 88:82–9. doi: 10.1097/ACM.0b013e31827647a0, 23165266 PMC3665769

[27] Almaiman AJM. Mentoring and the public health workforce: a scoping review. J Contemp Sci Res. (2015) 3

[28] Friedman DB Yelton B Corwin SJ Hardin JW Ingram LA Torres-McGehee TM . Value of peer mentorship for equity in higher education leadership: a school of public health focus with implications for all academic administrators. Mentor Tutor. (2021) 29:500–21. doi: 10.1080/13611267.2021.1986795

