# Supplementary material for: Advancing public health leadership through culturally centered and responsive research mentorship training in Nigeria
Source: Front Public Health. 2025 Dec 16;13:1611853. doi: 10.3389/fpubh.2025.1611853 (PMC12748261; doi:10.3389/fpubh.2025.1611853)
Supplement: Supplementary file 1 [file Data_Sheet_1.docx]

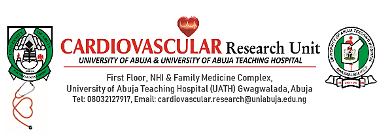

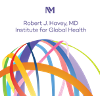

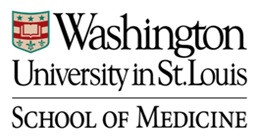

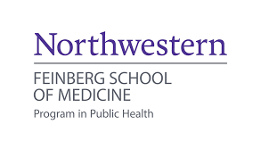


**AGENDA**

**CERTIN Mentorship Workshop**

**Venue: Rockview Hotel, Wuse II, Abuja**

***Dates: Thursday April 25 and Friday April 26th 2024***

***Funded by the Cardiovascular Research Training in Nigeria (CeRTIN) D43: Building capacity in Clinical Trials, Patient Centered Outcomes Research and Implementation Science . NIH/Fogarty International Center: D43TW011976***

The main objectives of this second mentorship workshop include:

1. Develop a team of faculty leaders and mentors in Nigeria who can provide mentorship and training for new cardiovascular investigators and sustain the cardiovascular research agenda.

2. Guide early career and fellows on how to navigate to scale the hurdles to the top.

3. Provide more senior investigators at the University of Abuja and strategic partners with skills and competencies necessary to mentor junior researchers and to develop a research workforce capable of conducting high-impact cardiovascular research at independent and team levels.25-26

4. Nurture a culture of institutional mentoring, competency-based frameworks, peer mentoring, cohort learning, and learner-centered pedagogy.

***Day 1***

| Session | Time | Session | Goals |
| --- | --- | --- | --- |
|  | 8:30 - 9:30 | **Registration; arrival; fill out name tags; table, pre-evaluation** |  |
|  | 9:30 - 10:00 | **Introductions** | Goals of the workshop  Name, institution, current role (mentor/mentee/both),  name your table |
|  | 10:00 -10:20 | **Session and community norms** | Community guidelines for discussion and sharing; thoughts added to by groups |
|  | 10:20 -10:45 | **Mentoring overview - frameworks, definitions, ecosystem, and models** | Provide overview of mentoring areas, frameworks, and model   - Domains: Supervision; Mentoring; Coaching; Sponsorship - Frameworks that describe Research, Interpersonal, Psychosocial and career, culturally responsive/diversity, Sponsorship - Models of mentoring including CeRTIN triad - Tools you can use |
|  | 10:45 -11:15 | **Break** |  |
|  | 11:15 -11:45 | **Small group,** | Setting intentions for the workshop and for mentoring   - What are your learning goals and outcomes? - Expertise you add to the room; asset-based recognition and sharing - Experience in mentoring and being a mentee - Experience in using tools: Mentoring journaling for reflecting, planning, recording, remembering - Something you are curious about   **Worksheet and instructions** |
|  | 11:45 -12:00 | **Selected groups report back** |  |
|  | 12:00 -12:30 | **Small group interaction, mentors and mentees combined** | Small groups fill out worksheet describing actions/activities within the different domains for mentoring, specifying the goals and outcomes  **Worksheet and instructions** |
|  | 12:30 -12:45 | **Selected groups report back** |  |
|  | 13:00 -14:00 | **Lunch** |  |
|  | 14:00 -14:45 | **Insights and strategies to effective mentoring in the local context** | Talk, questions and answers, and small group discussions on learning points |
|  | 14:45 -15:00 | **Open discussion** | Reflecting on power dynamics around positionality, career status, gender, age |
|  | 15:00 -15:30 | **Mentoring and mentee competency-individual work** | Self-assessment and reflections  **Mentor and Mentee Self-Assessment worksheet and instructions** |
|  | 15:30 -16:00 | **Hearing from the mentee panel** |  |
|  | 16:00 -16:15 | **Closing and complete Critical Incidents Questionnaire** | Critical Incidents Questionnaire worksheet and instructions |

***Day 2***

| Session |  |  |  |  |  |
| --- | --- | --- | --- | --- | --- |
|  | 8:30-9:00 | Arrival, meet and greet; find new seats |  | | |
|  | 9:00-9:30 | **Brief introduction, reflections from the Critical Incidents Questionnaire** |  | | |
|  | 9:30-9:45 | **Setting expectations by mentors and mentees-what they are and how** | - What are the expectations for mentee and mentors? - How are expectations communicated? - What are the cultural factors which may influence? | | |
|  | 9:45-10:15 | **Individual-complete worksheet** | **Worksheet and instructions** | | |
|  | 10:15-10:30 | **Report back** |  | | |
|  | 10:30 - 11:00 | **Goal setting and monitoring** | - Setting goals and developing reflective planning, SMART goals, IDP - What are successful mentoring outcomes?   **Worksheet and instructions** | | |
|  | 11:00-11:15 | **Break** |  | | |
|  | 11:15-11:45 | **Sharing goal setting experiences and monitoring: Small group mixed mentor and mentee** | Group Review of IDP  Reflection activity – Small group-mentor/mentee   - Have you used IDPs? - Where have they been helpful? - What has been challenging? - What else would like to see in the IDP? - How you would you adapt the one shared? | | |
|  | 11:45-12:00 | **Report back** | Key learning points | | |
|  | 12:00-12:30 | **Ethical challenges in mentoring: Open discussion** | What are some possible ethical challenges in the domains of mentoring? Research, supervision  What might be ways to address? | | |
|  | 12:30-13:00 | **Giving effective feedback: Lecture** | Lecture  Neighbor discussions | | |
|  | 13:00-14:00 | **Lunch** |  | | |
|  | 14:00-14:40 | **Providing mentorship for early career persons** | Online lecture | | |
|  | 14:40-15:00 | **Role play: practicing feedback conversations**  **3 people in a group: Divide mentors from their mentees** | Role play worksheet  Small group activity  **Workshop and instructions** | | |
|  | 15:00-15:20 | **Report back** | Key learning points: Techniques mentors/mentees can practice | | |
|  | 15:20-16:00 | **Reflections on my experience in mentoring in the local context** | Lecture | | |
|  | 16:00- 16:15 | **Post-evaluation**  **Wrap-up and summary** |  | | |

Resources:

<https://www.postdocacademy.org/mentoring/postdoc-activities/>

Bennett B. Goldberg, Erasto V. Mbugi, Fatima Kyari, Sara E. Woods, Emmanuel Balandya, Denise Drane, Rifkatu Reng, Deodatus Kakoko, “Training in the art and science of facilitation to scale research mentor training in low and middle income countries,” Front. Educ. Sec. Higher Education Volume 8 – 2023. [doi: 10.3389/feduc.2023.1270480](https://doi.org/10.3389/feduc.2023.1270480)
