# Supplementary material for: Advancing public health leadership through culturally centered and responsive research mentorship training in Nigeria
Source: Front Public Health. 2025 Dec 16;13:1611853. doi: 10.3389/fpubh.2025.1611853 (PMC12748261; doi:10.3389/fpubh.2025.1611853)
Supplement: Supplementary file 2 [file Data_Sheet_2.docx]

**Individual Development Plan (IDP)**

An **Individual Development Plan (IDP)** is a tool for early career faculty to first reflect on and then communicate their training, research, career goals, professional development needs, and progress toward meeting these goals with their Mentor Team and Program Leadership. Faculty should schedule regular mentoring meetings and use the IDP to update their progress and reflect themselves:

- **Mentor Team**: XXX (Primary Mentor), YYY (Methodological Mentor), ZZZ (Clinical Mentor)
- **Quarterly Mentorship Meeting**
  - The Mentor Team Meeting should include the faculty member, Primary Mentor, Methodological Mentor, and Clinical Mentor, if relevant, and any additional mentors.
  - Meetings should be held in **about 3-4 times/year**.
  - Your IDP should be updated and shared with your mentors in advance of the meeting. The faculty member should use their section of the IDP to assess and reflect on the mentee’s progress,
  - The Faculty Member should schedule these meetings well in advance to assure mentor availability.
- **Individual Meetings with Mentors**: The Faculty Member should hold regular meetings with their mentors. Every 2-3 weeks can be helpful, even if only for a short meeting.

| **Quarterly Team Meeting Planner** | | |
| --- | --- | --- |
| **Date** | **Location** | **Attendees** |
|  |  |  |
|  |  |  |
|  |  |  |
|  |  |  |

- **Reflection and goal setting**: In the space below, reflect on and express your short term, medium term, and long term goals for your career. Link the three time points. They can be several or just a few years apart. Think about making your goals SMART – Specific; Measurable; Attainable; Relevant and Timely: A specific goal is clear; a measurable goal is one for which it is easy to define success; an attainable goal is within reasonable reach; a relevant goal aligned with your aspirations; and a timely goal has a time period well defined.

| Goal # | **Long term, ultimate goal** | **Medium term goal on path** | **Short term goal** |
| --- | --- | --- | --- |
| 1. |  |  |  |
| 2. |  |  |  |
| 3. |  |  |  |
| Mentor thoughts and reflection: | | | |

- **Desired Skills and Competencies:** Here, for each of the major goals listed above, list the skill or competency you feel you will need to achieve that goal.

| **Goal** | **Desired Competency or Skill** | **Plan for Obtaining the Competency or Skill** |
| --- | --- | --- |
|  |  |  |
|  |  |  |
|  |  |  |
|  |  |  |
| Mentor thoughts and reflection: | | |

- **Research Projects: Please add a new section for each research project.**

| Project Title/Topic: |
| --- |
| Purpose: |
| Your Role and responsibilities: |
| Progress and Update Since Last Meeting. Identify successes, failures, and roadblocks: |
| Self-assessment and Issues for Discussion: |
| Papers, presentations and their status (*under development, awaiting feedback from mentors, submitted, in press, etc.):* |

| **General Comments from Mentor(s):** |
| --- |

- **Secondary Projects:** *Describe any other research efforts (e.g., review articles, book chapters)*

| Project Title/Topic: |
| --- |
| Purpose: |
| Your Role and responsibilities: |
| Progress and Update Since Last Meeting. Identify successes, failures, and roadblocks: |
| Self-assessment and Issues for Discussion: |
| Papers, presentations and their status (*under development, awaiting feedback from mentors, submitted, in press, etc.):* |

| **General Comments from Mentor(s):** |
| --- |

- **Training (e.g., Workshops, Short Courses, Seminars):** For each training, link to a skill or goal from above.

| **Goal or Skill** | **Date** | **Title** | **Outcomes and learning** |
| --- | --- | --- | --- |
|  |  |  |  |
|  |  |  |  |
|  |  |  |  |
|  |  |  |  |
|  |  |  |  |
|  |  |  |  |

- **Professional Meetings**:

| **Name of Meeting** | **Date** | **Abstract Submitted?** | **If Yes, Title of Abstract** | **Status and Type of Presentation** |
| --- | --- | --- | --- | --- |
|  |  |  |  |  |
|  |  |  |  |  |
|  |  |  |  |  |

- **Grant Applications:**

| **Title/Topic** | **Funder and**  **Type (e.g., F, K)** | **Due Date** | **Status** | **Issues for Discussion** |
| --- | --- | --- | --- | --- |
|  |  |  |  |  |
|  |  |  |  |  |
|  |  |  |  |  |

- **Presentations:**

| **Date** | **Topic** | **Issues for Discussion** |
| --- | --- | --- |
|  |  |  |
|  |  |  |
|  |  |  |
|  |  |  |
|  |  |  |
|  |  |  |

- **Clinical/Laboratory Work :**

Estimated clinical/laboratory responsibilities (e.g., hours per week or percentage of time):

- **Additional Issues for Discussion at Mentoring Meeting**:
